# Healthy competition drives success in results-based aid: Lessons from the Salud Mesoamérica Initiative

**DOI:** 10.1371/journal.pone.0187107

**Published:** 2017-10-27

**Authors:** Charbel El Bcheraoui, Erin B. Palmisano, Emily Dansereau, Alexandra Schaefer, Alexander Woldeab, Maziar Moradi-Lakeh, Benito Salvatierra, Bernardo Hernandez-Prado, Ali H. Mokdad

**Affiliations:** 1 Institute for Health Metrics and Evaluation, Seattle, Washington, United States of America; 2 El Colegio de la Frontera Sur, Barrio Maria Auxiliadora, San Cristóbal de las Casas, Chiapas, MÉXICO; University of Sydney, AUSTRALIA

## Abstract

**Objectives:**

The Salud Mesoamérica Initiative (SMI) is a three-operation strategy, and is a pioneer in the world of results-based aid (RBA) in terms of the success it has achieved in improving health system inputs following its initial operation. This success in meeting pre-defined targets is rare in the world of financial assistance for health. We investigated the influential aspects of SMI that could have contributed to its effectiveness in improving health systems, with the aim of providing international donors, bilateral organizations, philanthropies, and recipient countries with new perspectives that can help increase the effectiveness of future assistance for health, specifically in the arena of RBA.

**Methods:**

Qualitative methods based on the criteria of relevance and effectiveness proposed by the Development Assistance Committee of the Organization for Economic Co-operation and Development. Our methods included document review, key informant interviews, a focus group discussion, and a partnership analysis.

**Participants:**

A purposive sample of 113 key informants, comprising donors, representatives from the Inter-American Development Bank, ministries of health, technical assistance organizations, evaluation organizations, and health care providers.

**Results:**

During May–October 2016, we interviewed regarding the relevance and effectiveness of SMI. Themes emerged relative to the topics we investigated, and covered the design and the drivers of success of the initiative. The success is due to 1) the initiative’s regional approach, which pressured recipient countries to compete toward meeting targets, 2) a robust and flexible design that incorporated the richness of input from stakeholders at all levels, 3) the design-embedded evaluation component that created a culture of accountability among recipient countries, and 4) the reflective knowledge environment that created a culture of evidence-based decision-making.

**Conclusions:**

A regional approach involving all appropriate stakeholders, and based on knowledge sharing and embedded evaluation can help ensure the effectiveness of future results-based aid programs for health in global settings.

## Introduction

Results-based aid (RBA) is a financial assistance model involving a contract between a donor and a national government where either a portion of or all donor funds are disbursed based upon performance or predetermined targets [1–4]. RBA for health emerged in 2010 with Gavi, the Vaccine Alliance, as a pioneer for this kind of assistance [5], and replacing its performance-based Immunization Services Support grants that were in place between 2000 and 2010. With the novelty of the RBA approach for health, its success has not yet been confirmed but it is seen as a promising approach for aid agencies and philanthropies.

Very few RBA models for health exist, and among these is the Salud Mesoamérica Initiative (SMI). SMI is a public-private partnership funded by the Bill & Melinda Gates Foundation, the Carlos Slim Foundation, and the Spanish Agency for International Development Cooperation, and administered by the Inter-American Development Bank (IDB) who also provides countries with tailored technical assistance as needed. It aims to reduce maternal and child mortality and health inequities for the poorest populations in seven Central American countries—Belize, Costa Rica, El Salvador, Guatemala, Honduras, Nicaragua, and Panama—and the southern State of Chiapas, Mexico. Specifically, SMI aims at 1) reducing by 15 percent infant mortality among the poorest 20 percent of the region’s population; 2) providing health services to 260,000 poor children to reduce chronic malnutrition; 3) reducing by 15 percentage points the rate of anemia among children under two years in seven of the eight countries in the region (in Chiapas, 10%); 4) ensuring that 90 percent of children under two years among the poorest 20 percent of the population complete their vaccination schedules; and 5) increasing by 50 percent births attended by skilled personnel, in order to reduce deaths of mothers and newborns.

SMI is based on a participatory effort from the countries above who put together documents known as the Master Plans, and which consisted of cost-effective interventions to reduce maternal and child mortality. A cost-analysis was later performed to determine the possible change, in a set of indicators, that could be achieved based on the selected interventions, and by country. For example, an indicator can be a percentage point increase in number of pregnant women who received at least four antenatal care visits, or the percent of health care centers that have the necessary equipment to provide basic child care [6].

**Fig 1:**
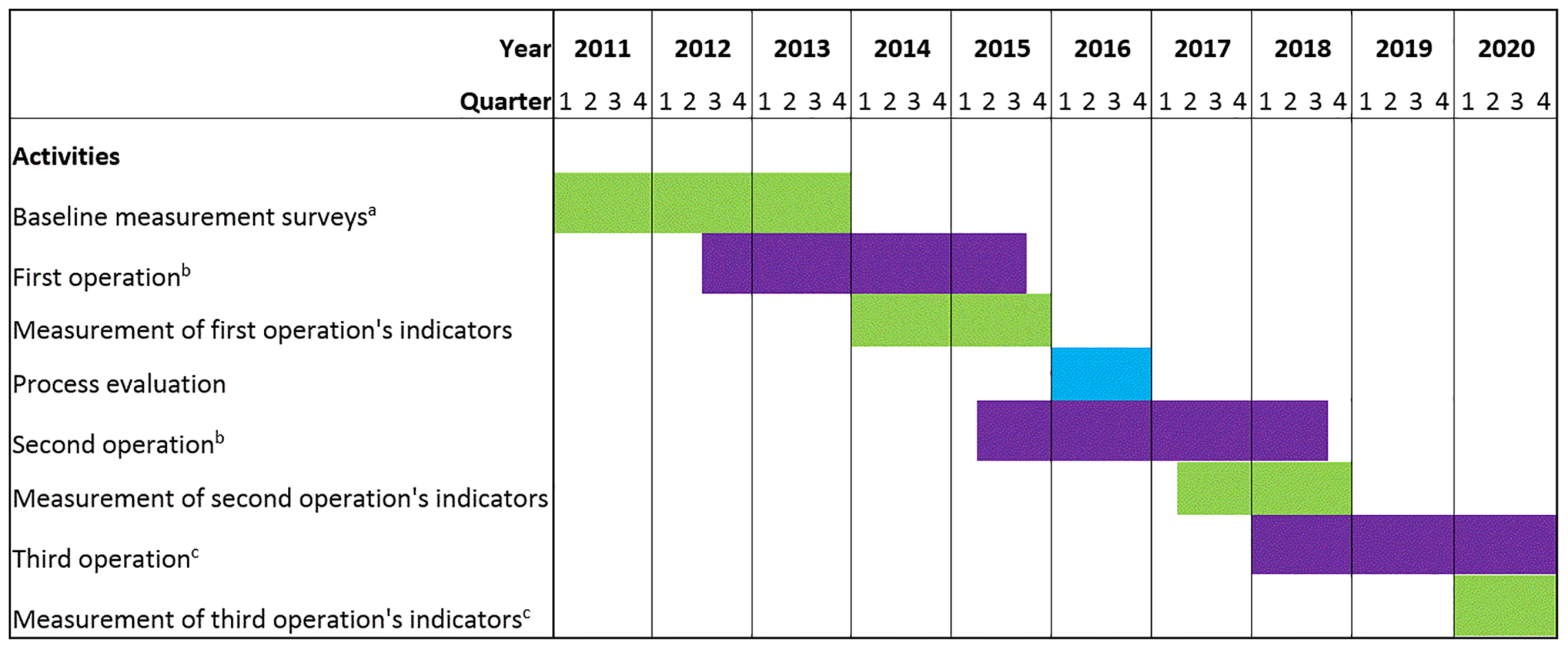
Timeline of Salud Mesoamerica Initiative’s operations and respective indicators’ measurements. ^a^: baseline measurements were conducted in 2011 only in El Salvador. ^b^: on average operations are 24 months but do not start at the same time in all countries and hence might appear longer. ^c^: dates for the 3rd operation and its respective indicators’ measurements are not final at this time.

SMI is a three-operation strategy. Fig 1 represents the timeline of SMI operations and indicators’ measurement. The first operation’s targets were focused on inputs for health systems, while those of the second and third operations are focused on service delivery and demand generation. Participating countries negotiate the interventions and respective targets with IDB, and receive tailored technical assistance to implement the selected interventions. Through SMI, participating countries receive 50% of the cost of an operation from funders and contribute the remaining 50% themselves. At the end of each operation, pre-defined target indicators are measured independently, and if 80% of these indicators are met, the country is awarded half of its contribution share back and permitted to continue on to the initiative’s next phase. Funds from this “performance tranche” can be used freely within the health sector at the country’s discretion [6].

SMI operations launched in the summer of 2012, and by the beginning of 2014 measurement results for the first operation were available from all eight countries. Five of the eight participating countries successfully reached the target indicators for the first operation. Despite not being totally successful, the remaining three countries made significant improvements on their indicators as well [7]. Specifically, Chiapas did not meet the targets of the first operation. However, targets were eventually reached following a six-month improvement plan. This success in meeting pre-defined targets is rare in the world of financial assistance for health. While most success of global health assistance has been the raising of significant amounts of new resources, a clear impact has often been hard to prove for separate initiatives or programs [8], and SMI presents a learning opportunity for the arena of financial assistance for health. This has been envisioned since the early stages of SMI design when process evaluations were planned in order to identify the factors that promoted or affected the initiative’s success [9]. We conducted a process evaluation of SMI. Within this evaluation, we investigated the influential aspects of SMI that could have contributed to its effectiveness in improving health systems, with the aim of providing international donors, bilateral organizations, philanthropies, and recipient countries with new perspectives that can help increase the effectiveness of future assistance for health, specifically in the arena of RBA. This study considered the overall initiative for all participating countries for most of the investigation, and focused on Chiapas, Mexico for the specific topic of implementation.

## Methods

To answer the question on SMI’s influential components, we developed a list of topics and questions based on the criteria of relevance and effectiveness proposed by the Development Assistance Committee of the Organization for Economic Co-operation and Development. Our qualitative methods were based on document review (DR), key informant interviews (KII), a focus group discussion (FGD), and a partnership analysis, all framed in a grounded theory approach. Briefly, grounded theory is a qualitative research method used to develop theory from data collected systematically and analyzed. The theory developed is not static but evolves continuously with the collection and analysis of data [10]. This study received institutional review board (IRB) approval as a non-human subject research determination from the University of Washington and partnering data collection agency, El Colegio de la Frontera Sur. Verbal consent was obtained from study participants prior to beginning of interviews.

### Document review

An exhaustive document review was conducted to refine the evaluation questions, create the topic guides for key informant interviews, identify potential key informants, triangulate interview findings, and account for information that was not easily remembered by relevant key informants. Documents were provided by IDB’s SMI Coordinating Unit, as well as obtained from publicly available sources online. The documents included the original initiative proposals; operating regulations; regional master plans for specific health areas; national norm documents; operational plans and strategies; annual reports; quarterly reports; documents about specific initiative areas such as dashboards and policy dialogue; descriptions of the indicators and targets set for the initiative; monitoring and evaluation documents; budgets; studies and needs assessments conducted for the initiative; meeting minutes; theories of change; and presentations related to the initiative.

### Key informant interviews

*Topic guide development*: Structured topic guides for key informant interviews were developed following the document review, exchanges with stakeholders, and a fact-finding mission conducted in Chiapas, Mexico. We sought to identify the factors that impact key stakeholders’ decisions, allocation of resources, and effectiveness. The topic guides covered the components of the SMI theory of change. These focused on issues related to the planning, design, and implementation of SMI, as well as results, efficiency, and the lessons learned thus far. KII topic guides were tailored specifically to each of the donors, IDB, the Project Coordinating Unit in Chiapas, Management Sciences for Health, Secretaria de Salud of Mexico (SSA), Instituto de Salud del Estado de Chiapas (ISECH), jurisdiction leaders in Chiapas, and health care providers in SMI and non-SMI areas.

*Sample selection*: Two main groups of key informants (KIs) were purposively sampled. The first group included decision-makers from all SMI partner organizations. These organizations included funders of SMI (global key informants), IDB, ministries of health (national key informants), and the Chiapas ministry of health (local key informants). The second group consisted primarily of programmatic actors such as managers of health care facilities and health care providers.

The SMIPE team compiled a list of key stakeholders who played a crucial role in the initiative, either as KIs or by providing support with the process of the initiative. KIs were identified through previous work on the SMI quantitative evaluation, through document review, and as suggested by SMI stakeholders.

KIs were identified based on the following criteria:

- Their role in the design and funding of SMI in general
- Their role in the design, planning, and implementation of the SMI plan for Chiapas
- Their role in the ministries of health in SMI countries
- Their role in SMI with regard to Mexico

*Data collection*: Interviews were conducted by the researchers leading the study. Respondents were informed that their responses would be kept confidential and anonymous. Interviews were conducted in the preferred language of the key informant, either English or Spanish. Interviews lasted between 60 and 90 minutes, were audio-recorded and transmitted to the study team using a secure server. CEB and BHP separately interviewed most participants of the first group face-to-face, in a private space at the participants’ place of work. Four participants were interviewed via Skype due to their location. CEB and BHP are males, and have completed PhD degrees in biological anthropology and psychology respectively, and are professors at the University of Washington. Participants received interview requests via email. The emails detailed the purpose of the study and the topics that will be discussed during the interviews. CEB and BHP were accompanied by a Bachelor’s level data analyst who took note during the interview. When CEB interviewed Spanish-speaking participants, an interpreter was present and provided in-time interpretation. The topic guide for this first group is available as S1 Text.

Participants of the second group were interviewed face-to-face, in a private space at their place of work, by a team of eight researchers from El Colegio de la Frontera Sur in Chiapas. Only one of the researchers was a male, and all researchers had a least a bachelor’s degree in social sciences and received a training on the study tools and procedures prior to data collection. Participants of the second group were contacted by the research team prior to the interviews who carried an introductory letter detailing the study from the Chiapas ministry of health. The topic guide for this second group is available as S2 Text.

### Focus group discussion

*Topic guide development*: A topic guide was developed in order to capture how regional ministries of health perceived SMI’s design and implementation. The topic guide for this focus group discussion is available as S3 Text.

*Sample selection*: The focus group conveniently included relevant stakeholders representing ministries of health from six Mesoamerican countries, who were in Panama for the Latin American and Caribbean Conference 2016: Honduras, Guatemala, Costa Rica, Belize, El Salvador, and Panama.

*Data collection*: The focus group was conducted by CEB who took note during the discussion. The focus group was conducted in English in the presence of an interpreter who provided in-time interpretation as most participants spoke only Spanish. Participants received interview requests via email. The emails detailed the purpose of the study and the topics that will be discussed during the interviews. Participants were informed that their responses would be kept confidential and anonymous to the extent possible. The focus group lasted about 90 minutes and was audio-recorded. Only one interview was repeated due to failure of audio-recording.

### Partnership analysis

*Partnership tool design*: We used the PARTNER (Program to Analyze, Record, and Track Networks to Enhance Relationships) tool, described elsewhere, to demonstrate how members are connected, and measure the levels of trust in the network [11]. The tool asked key informants representing the organizational stakeholders of SMI to report their perception of partner organizations. Surveyed partners also responded to multiple-choice questions regarding their views on SMI’s objective, its success, and the aspects of collaborative work that propel the success it has achieved so far.

*Sample selection*: donor organizations, IDB, technical assistance partners, evaluation partners, ministry of health officials, and the evaluation organization

*Data collection*: We administered the PARTNER questionnaire in both English and Spanish to a broad set of SMI stakeholders and collected data on paper and online.

### Data analysis

Audio recordings of the interviews were transcribed verbatim. Interviews conducted in Spanish were translated to English. All transcripts were manually coded and analyzed for their content through recursive abstraction by a PhD and a post-doctoral students in health metrics and evaluation, and two data analysts holding bachelor’s degrees in social sciences. All four attended a training on recursive abstraction at the start of data analysis. In short, recursive abstraction is a qualitative data analysis method consisting of the following steps:

1. Transcribed data are grouped by topic and respondent to form a matrix.
2. Portions of comment that are relevant to the topic at hand are extracted and formed into code-like sentences.
3. Coded responses from various stakeholders within each respondent group are analyzed together to generate brief summary narratives. Agreement between different audiences and document review, as well as level of shared knowledge among respondents, are taken into consideration.

Themes, each covering a number of codes, were derived from interview and focus group data, and were used to answer the evaluation questions. Data analysis was conducted in parallel to data collection to monitor data saturation which was reached towards the third participants in each of the different groups. Given time restraints, the size of the sample, and the length of transcripts, these were not returned to participants for comments, or corrections.

## Results

During May–October 2016, we interviewed 113 key informants regarding the relevance and effectiveness of SMI (Table 1). Six donor, ISECH, and SSA representatives were contacted but not reached for an interview from a total of 62. Of 59 health care providers approached for an interview, only a couple refused to participate in the study without giving a specific reason. Four major themes emerged relative to the topics we investigated, and covered the design and the drivers of success of the initiative. Each theme was parent to a number of codes or patterns.

**Table 1:** Key informants of the Salud Mesoamerica Initiative process evaluation.

Study audience	Key informants
SMI donor representatives	11
IDB + Project Coordinating Unit + Management Sciences for Health	13
Regional MOH representatives (outside of Mexico)	9
ISECH (including jurisdiction leaders)	17
SSA	6
Health care providers—SMI communities	45
Health care providers—non-SMI communities	12
**TOTAL**	**113**

### Theme 1: Robust design aligned with countries’ priorities and sense of a real partnership

The document review, KII, and focus group discussion showed that the RBA model was designed between donors and IDB. However, for each operation, target indicators were set in negotiation with participating countries. Strategies were also selected with countries’ participation through the creation of master plans. These are a catalog of cost-effective proven activities that can be implemented through SMI. The specific details of activities and timelines were agreed upon with implementing bodies from local ministries of health, such as directors of divisions of maternal and child health, jurisdiction health leaders, and even health care providers in some instances. The last word in decision-making regarding each of the operations was always up to participating countries. For instance, in Chiapas, leaders of health jurisdictions transmit the needs of their communities, and the potential solutions, to ISECH, who in turn negotiate interventions with IDB.

“*We participated from the election of communities which were the 17 municipalities*. *There are 18*, *but 17 actually were the ones participating*, *and they're completely indigenous*. *We were trying to have all of those which are the most marginalized*, *the ones that are very*, *with an extreme level of poverty*. *So that was a situation where they asked for our opinion*, *and of course*, *I said that all of them should be included… For instance*, *basically the indicators; meaning where we were aiming for*, *which are three indicators*, *the ones that really have an impact on us*: *Maternal mortality*, *perinatal mortality*, *and providing care to the poorest cities or towns*. *That's—That one includes nutrition for newborns and child—children under five years of age*.”

Health leader from Chiapas, Mexico.

In parallel, health jurisdiction leaders keep health workers informed on the initiative and its operations.

“*The jurisdiction has shared with us the objectives of SMI and we are trying to work on these objectives*, *working towards the millennium goals*. *The SMI is about the fourth and fifth millennium development goals*.”

Health worker from a facility in Chiapas, Mexico.

“*The first stage determines the minimum equipment and supplies needed without breaking the supply chain*. *For example*: *ophthalmic chloramphenicol—oxytocin*, *those things*, *right*? *We need those things available and prevent breaking the supply chain in all shifts*. *This is an evaluation that we use*. *In his strategy*, *the Doctor assigns two people*. *One responsible for the warehouse and modification*. *And the other is the liaison with the pharmacy*, *since the pharmacy is subrogated*. *Meaning*, *we don't purchase directly*, *we subrogate and depend on the pharmacy*. *We have changed pharmacy three times in this time*, *thus*, *we have had to adjust to all the changes and try to maintain the supply chain*. *We're doing good so far*.*”*

Health worker from a facility in Chiapas, Mexico.

**Fig 2:**
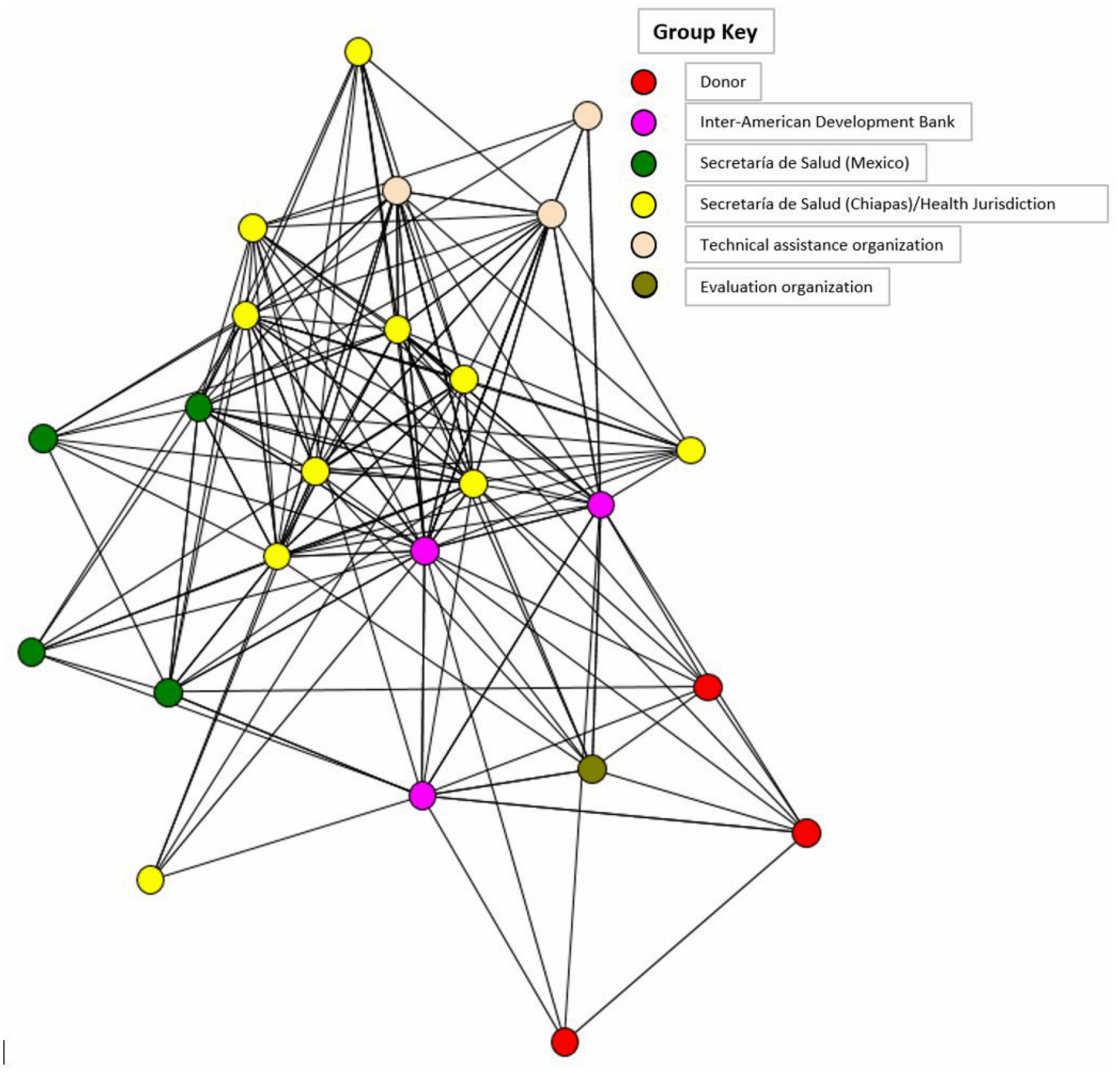
Salud Mesoamerica Initiative network for Chiapas.

Despite the engagement of all levels, many felt the lack of a true partnership in the beginning due to the initiative’s strict rules about meeting target indicators. Over time, this situation changed and SMI partnership grew stronger as countries’ perspectives became more valued and a shared vision based on mutual trust developed as shown by KII and the partnership analysis. As observed in Fig 2, and in the specific case of SMI in Chiapas, all players are strongly connected within the SMI network, with the greatest connectedness seen at the most local level.

Both the document review and KII indicate that SMI is based on a flexible theory of change with stable goals of reducing maternal and child mortality and health inequalities among the poorest populations in Mesoamerica. This theory of change evolved from linear to multidimensional to mirror the development of the initiative’s complex design, and incorporate the multitude of model components that dictate SMI’s strategic and operational plans. Each country operation is based on a specific theory of change as well. SMI is perceived by countries’ stakeholders as making huge achievements in health systems in the area.

SMI goals, originally designed to align with the Millennium Development Goals, are well aligned with national health plans from participating countries.

“*In the case of Guatemala*, *well*, *for a long time the maternal care*, *maternal and child care will continue to be a very important topic*. *And that is what the initiative focuses on*. *So up to a point there is an alignment in terms of priorities*.”

—High level representative attending the focus group discussion at the Latin American and Caribbean Conference 2016.

“*I believe there are two elements where the initiative has aligned itself very well with the country initiatives*. *And it aligned to improve—to empower the country initiatives*. *And the two elements that I’m thinking of right now is in the decentralization process*, *as we call it in Honduras*, *that has consisted in the past few years in separating the service provision that used to the be the responsibility of the secretariat itself*, *and for the past ten years*, *give or take*, *they’ve been transferring this power to other institutions different than the secretariat*. *This has been an initiative of the Honduras health secretariat*. *And SMI abided by that country decision*. *And with their strategy and co-financing*, *they’ve been able to empower that country initiative*. *The other one is the one that is an effort being carried out by the country for many years*. *It’s the reduction of maternal and child mortality*. *And the initiative is strongly aiming towards that goal to improve access of women and children to health services*, *as well as improving the response capabilities of the services to the mother/child*. *And initiative really coupled very well to the processes that the secretariat already had in place empowering them actually*”

—High level representative attending the focus group discussion at the Latin American and Caribbean Conference 2016.

“*The actions generated by the SMI are similar to those in the specific action plans of the national health plan*. *The difference is that the SMI poses very clear*, *specific*, *concrete actions that*, *in my opinion*, *have helped Chiapas define very clearly were the resources go to*. *I think the objectives of the SMI align with those of the National Health Plan*. *Perhaps the SMI objectives were defined before the National Health Plan*, *but the objectives are the same*.*”*

—High level representative from the Ministry of Health in Mexico.

To achieve SMI goals, a multiplicity of assessments, verified through document review, were conducted, including cost-effectiveness studies, access barriers, and secondary data analysis from pre-existing surveys in each of the countries. A set of strategies were selected based on these assessments to address the identified bottlenecks through a systematic approach. Integration between the strategies and activities, a key aspect of SMI, increased between the first and second operation as pointed by KI. Despite the multiple assessments done, a baseline round of household surveys was conducted following the selection of strategies, which is counterintuitive and a step out of order as reported by most key informants. Nevertheless, the results of these surveys were mostly in line with the needs or bottlenecks identified in the other assessments.

### Theme 2: Regional model creates peer pressure

SMI’s regional approach had the unintended outcome of sparking a competition between participating countries toward meeting target indicators under positive peer pressure. This sense of competition was reported by KIs from among donors, IDB, regional MOH representatives, ISECH, and SSA as the strongest factor in pushing countries to achieve results. Countries feared for their reputation among their neighboring countries.

“*We mobilize everybody in terms of this objective and this goal*. *So I believe that—I don’t really know how to describe it*. *But in that sense*, *we feel like we’re taking an exam and we want to pass*. *We don’t want to look bad as a country*. *We want to truly show and prove that we can make our best efforts and we can achieve the results*. *So this mechanism is what—well*, *this allows us to do things that are not as clear with other projects…*. *And the fact that it’s also with other countries*: *it adds an additional pressure on top of that additional pressure*. *Because we don’t want to end up looking bad*. *So we’re always aware and pending*.”

—High level representative attending the focus group discussion at the Latin American and Caribbean Conference 2016.

Specifically in the case of Chiapas, Mexico, not meeting the target indicators for the first operation led to huge efforts through an improvement plan to successfully meet these indicators, despite this additional plan not being funded prior to implementation.

“*That was a big change and the fact that they failed so badly was also*, *I think*, *the part of the drive so that they decided to change the way they were approaching things and*, *of course*, *there was also a federal response to be closer*, *to work more closely with the state of Chiapas and supervision actions*. *I think everyone had a role to play*, *but I think the change at the state level*, *the jurisdictions*, *was the key to this*.*”*

—High level representative from the Ministry of Health in Mexico.

At the same time, this approach also allowed for an inter-country learning and support platform that would not have been possible had the initiative been implemented independently in the participating countries, or in countries from different regions. Third, the regional approach allowed for a more efficient use of funds invested in purchases as emphasized by KI. Negotiations through COMISCA meant economies of scale for drug, medicine, and supply procurement, reportedly resulting in reduced prices. Fourth, relevant KIs reported that the approach increased efficiency in the use of technical assistance across countries from regional to field units.

### Theme 3: The results-based aid embedded evaluation creates a culture of accountability

The RBA model reportedly created a culture of accountability given the design-embedded evaluation component in SMI. Countries were aware that measurements would take place at the end of each operation, and that increased the sense of urgency to achieve results. Specifically in Chiapas, this culture was strengthened following the failure to meet targets in the first round. With the improvement plan, accountability trickled down to the level of health workers, who developed the habit of routinely checking supplies and ensuring shortages were avoided.

“*I check that all the materials and equipment are available*, *otherwise I raise all necessary reports to the manager or maintenance areas*. *It is a multidisciplinary team*, *so if one doesn’t work*, *the rest don’t work either*. *So I make sure that everything necessary is available and the staff trained*.”

Health worker from a facility in Chiapas, Mexico.

Countries also developed strong and diverse monitoring systems for SMI activities and indicators to avoid surprising results from the external evaluation.

“*This allows us to mobilize the decision-makers from the health secretariat around this financing*. *We’ve commented—I don’t know*. *I don’t know actually*. *Because we all have to reduce maternal and child mortality and we have several projects aiming at that*. *The financing with the initiative is not the biggest financing the country’s receiving right now*, *so it’s not so much*. *Well*, *in fact*, *the financing*: *of course it supports and contributes to the country*. *But actually I believe that that feeling—well*, *nobody wants to fail*, *right*? *Nobody wants to*. *And we are being measured*. *So this actually pushes everybody to start moving and mobilizing*.*”*

—High level representative attending the focus group discussion at the Latin American and Caribbean Conference 2016.

“*Also*, *this self-assessment group was created*, *a group that is exclusively focusing in the assessment unit by unit to identify issues and solve them locally without waiting for this second assessment*, *external evaluation of the—and how the metrics instituted*.*”*

—High level representative from the Ministry of Health in Mexico.

Another component of the RBA model that was well-appreciated by KIs is the freedom to use the performance tranche. Countries could use this incentive for any health-related activity, and not necessarily in relation to SMI focus areas. The ability to use these funds anywhere in the health sector also played a motivating role for participating countries.

“*Some feedback that we’ve received from ministers of health is that though that amount is small*, *particularly the performance bonus*, *the part that is*, *as long as it’s spent within health*, *kind of unconditional in the ways in which they can allocate it*, *we’ve heard that that’s actually very valuable because their budget is almost completely fixed*. *Costs around human resources or other things where they can’t*, *they have no flexibility in how they allocate*. *So though it's small*, *we’ve received message that that small amount that they can direct as they see fit is highly valuable*. *So in so far as that may be connected to the motivation or kind of how that has potentially driven behaviors within the government to execute against the program*, *it seems like it has helped*.”

—High level representative from IDB

### Theme 4: SMI has fostered an experience-based learning environment

SMI’s knowledge-sharing environment is evidenced by the monitoring of implementation and progress, sharing of lessons across countries and localities, and use of evidence to make decisions. This can be seen both in the initiative’s documents and confirmed by KI. Ministers of health from SMI countries meet regularly to share experiences with, draw lessons from, and benchmark against each other countries. SMI has been guided by evidence from its early stages; several sources of information have been deployed to enhance the initiative, and the generated information has reportedly been used at every level. Beyond knowledge, this has created a culture of evidence-based decision-making. Country-specific evidence packages including barriers studies, cost-effectiveness studies, and secondary data analyses have been used to inform selection of strategies. Baseline measurement surveys have been used to set target indicators and a reference for comparison of following surveys. A dashboard has been developed to track country indicators. Information generated from the dashboard is being used by IDB and countries to track progress and ensure that operations are being implemented according to timelines. With the proven effectiveness of information tools developed in SMI areas, these tools are being disseminated to non-SMI areas.

“*The other detail in the day-to-day life in the hospital*, *for example—the periodic measurements carried out by the initiative have allowed us to modify clinical care processes*. *For example*, *the immediate postpartum care*: *that allowed us to realize that it was not the best*, *that it did not meet the requirements*, *the necessary quality requirements to ensure that the woman was receiving an adequate care in the immediate postpartum*. *So all the hospitals that benefited from the initiative already have a clinical guideline for postpartum care*. *Just to mention a simple example of how we’ve been able to use the data gained to modify internal processes in the hospitals*.*”*

—High level representative attending the focus group discussion at the Latin American and Caribbean Conference 2016.

“*From there on*, *well*, *that information is very valuable for us*, *and information we did not have*. *It has forced us somehow to review and revisit our information system*, *in fact to be able to provide follow-up on to do the indicators that we tried to—well*, *we actually didn’t try*. *And actually*, *it’s useful for us to know what’s going on*. *It’s results-based financing*, *and periodically we have to see what is happening*. *And we had not disaggregated the information as we have it now through the initiative*. *This allows us to focus on the service network or on the hospitals that have the most problems where we have to focus our efforts stronger*.”

—High level representative attending the focus group discussion at the Latin American and Caribbean Conference 2016.

## Discussion

This is the first study to comprehensively evaluate the process of an RBA for health like SMI. Specifically in this manuscript, we were able to document the drivers of SMI’s success, which we hope will help fill the need to identify methods that can increase the effectiveness of global health initiatives.

SMI is a pioneer in the world of RBA in terms of the success it has achieved in improving health system inputs following an initial 18-month operation, signaling a high potential for success for upcoming operations focused on service-delivery demand generation [7]. This success is due to 1) the initiative’s regional approach, which pressured countries to compete toward meeting targets, 2) a robust and flexible design that incorporated the richness of input from stakeholders at all levels, 3) the design-embedded evaluation component that created a culture of accountability among participating countries, and 4) the reflective knowledge environment that created a culture of evidence-based decision-making.

Many assistance programs have taken a regional approach in the past two decades, but these have been limited to the domains of development and security. For instance, European assistance to Central Asia focuses on building networks and partnership through a regional approach in order to fight against international crime, reduce poverty and increase living standards, and promote good governance and economic reform [12]. Similar objectives have been addressed since 2008 by the US Department of State, through a similar approach in the same region [13]. Regional aid-for-trade initiatives are known to increase cost-effectiveness in helping countries achieve their development goals [14]. This is still unknown vis-à-vis financial aid for health. SMI is the first RBA program to address health through a regional approach. The funds provided by SMI are not enormous and represent a tiny fraction of expenditure on health by governments in SMI countries. Hence, the reward itself, even if it did have some influence, could not have been motivating enough to drive SMI’s success. Indeed, the total cost of the first operation ranged from $1.3 million– 1.2% of total health expenditure—in Belize, to $11.7 million in Guatemala– 0.3% of total health expenditure[15,16]). However, the shared history, geography, and culture nurtured a peer-pressure environment among SMI countries and drove a healthy competition toward achieving results as noted in one of the quotes under the third theme. The case of Chiapas confirms this hypothesis further as Mexican stakeholders did not want to look less capable than their neighbors. This healthy competition through a regional approach might be a novel key to future global health assistance programs. Indeed, experience from other sectors shows that competition can be a strong motivator for success, even between different localities within the same country. For example, the District Development Facility (DDF) in Ghana is an RBA pilot for fiscal decentralization that started in 2008. This program has been showing signs of success as competition between districts has pushed local government to perform, increasing interaction between district administrations and local citizens, and therefore strengthening local accountability structures[17].

Klingebiel & Janus argue that RBA driven only by donors, or focused heavily on one measurable result, can lead to an increased risk of adverse incentives and non-systematic strategies[17]. The authors argue as well that success of RBA relies heavily on partner countries being in charge of the overall process in order to design RBA programs that are adapted to local contexts. SMI has been designed with appropriate input from all levels of stakeholders, from donors to local health jurisdiction leaders. The design of the initiative, its theory of change, and the rules of the game were set between donors and IDB. Further, the design of SMI has sought input from participating countries since its early stages, not only for strategy selection, but also for target indicators and performance level determination. The last word in decision-making has always been up to each of the countries with regard to their individual operations, and based on internal decisions between the different levels of the health system, including jurisdiction leaders who represent their local communities. Assessment of results has also relied on ten indicators in each of the countries, not just one, and these are directly related to the activities implemented in each operation. Moreover, no adverse incentives have been noted through SMI, which has been increasingly taking a systematic approach by integrating its strategies and activities.

SMI’s theory of change considers evaluation at the core of the initiative. Theoretically, prospective evaluations are preferred as baseline data can be collected to establish pre-program measures, as in the case of SMI. These baseline data describe both beneficiaries and control groups prior to the initiative and allow better target setting. Defining these targets during the initiative’s planning stage focuses the evaluation and the program on intended results. In prospective evaluations, control groups are determined early on, prior to implementation, and both control and treatment arms are observed over time[18].The prospective nature of SMI’s evaluation not only provides measurement of the results, but enables SMI’s partners to identify implementation bottlenecks along the way and devise solutions and corrective actions on the spot, as well as terminate a country’s participation if it does not meet targets twice. The knowledge that targets had not been met as soon as the end of the first operation in Chiapas opened the door for an action plan and allowed a correction of the course that the initiative was taking. Moreover, the benefits of SMI’s embedded evaluation extend even further as it fosters accountability. An accountability framework is created whenever an actor is required to justify its performance, a forum has the opportunity to question and judge this performance, and the actor faces consequences based on this judgement[19]. In SMI, countries are the actors, and funders are the forum. This goes beyond the concept of responsibility that most initiatives of assistance for health rely on, and where a recipient of aid is only expected to act ethically based on their moral judgment[20]. Indeed, the measurement surveys following the first operation and their use in determining the future of each country in SMI confirmed the intent of measuring results through SMI. This has contributed significantly to a culture of accountability where beneficiaries have recognized the importance of meeting targets. Along with the regional model, the evaluation component strengthened the competitive aspect, as countries knew they might be compared to other countries in the region.

The success of SMI’s knowledge-sharing environment can be explained by several factors as noted in the work of Holdt Christensen, 2005 and Ipe, 2003. First, knowledge-sharing is, and is perceived as, equitable for all stakeholders[21]. For instance, while the primary use of the household and health facility surveys is to measure progress, the data generated are freely available to countries, who can use them for other purposes. This reciprocity increased knowledge-sharing as stakeholders recognize that sharing their knowledge increases their added value [22,23]. Second, in knowledge-exchange forums, such as meetings of Ministers of Health, countries share lessons learned from activities that have not yet been implemented in other countries, and get to learn about ones they are yet to implement. These meetings are facilitated such that the tacit knowledge is simplified and directly communicated from the main stakeholder in each country[24]. Furthermore, the majority of the knowledge shared through SMI is explicit in nature. Due to the electronic data-capture methods used, SMI survey and dashboard data are easily stored, managed, and transferred across time and stakeholders[25]. Third, the knowledge-sharing environment created by SMI has blocked stakeholders from withholding the knowledge they possess from other actors in order to maintain a competitive advantage[26]. Hence, sharing decisions has been more positive than not[27]. Fourth, opportunities to share knowledge through SMI have been both formal, such as results dissemination meetings, and informal, through technical assistance or training programs[28]. Fifth, SMI has encouraged a culture of knowledge-sharing by making this practice a core component of its theory of change. This theory informs how knowledge would be created, shared, and used within the initiative[28].

Our study also identified some negative aspects that prevent success, such as an inability to operate effectively in the face of social problems in countries. The second operation has been facing social unrest in Chiapas, Mexico, which delays implementation significantly. At the same time, the state is going through a deep financial crisis that threatens to prevent Chiapas from securing its counterpart of the cost of the second operation. These factors might prevent the state once again from reaching SMI target indicators for the second operation. However, many risk mitigation strategies are being adopted to ensure the operation’s success. First, the initiative is focusing on local activities that do not require state involvement as transport around Chiapas has been heavily affected by roadblocks and teachers’ demonstrations. Second, with high-level staff turnover, the SMI coordination unit meets with newly hired directors to ensure commitment and continuation of the initiative activities. Third, technical assistance has been increased to help local teams make up for delayed implementation. Here, we can emphasize the importance of this qualitative investigation in uncovering such obstacles. The indicators’ measurement surveys that are already underway have been tailored to capture the effect of these obstacles, as well as the steps that have been taken to mitigate these obstacles.

This study has several limitations. First, memory bias and turnover of staff involved in SMI is a key limitation in the investigation of earlier periods of SMI. Hence, we relied on available documents to account for information that was not easily remembered by relevant key informants. Second, while data for the partnership analysis were collected from a broad range of regional organizations related to SMI as a whole, the lack of response from partners outside the operation in Mexico biased the regional network of collaborators. For this reason, we restricted the results to the network of partners around SMI in Chiapas. Last, we cannot ignore a social desirability bias. KIs were aware that the information collected would be for the evaluation of the initiative. Although confidence was established and many reported limitations and problems of the initiative, some may have emphasized the positive results more. Nevertheless, the initiative’s success in reaching its targets so far supports the positive findings reported here.

So far, SMI has proven to be a novel RBA with unprecedented improvements in strengthening health systems inputs. Its regional approach based on knowledge-sharing and embedded evaluation can help ensure the efficiency and effectiveness of future financial assistance for health in global settings. The results from the remaining operations will provide evidence for whether SMI will be successful beyond inputs, in strengthening services and increasing demand with the aim of reducing maternal and child mortality and health inequities.

## Supporting information

S1 Text Topic guide for first group of respondents including funders of SMI (global key informants), IDB, ministries of health (national key informants), and the Chiapas ministry of health (local key informants). (DOCX)

S2 Text Topic guide for second group of respondents including programmatic actors such as managers of health care facilities and health care providers. (DOCX)

S3 Text Topic guide for third group of respondents including representatives of ministries of health from six Mesoamerican countries, who were in Panama for the Latin American and Caribbean Conference 2016: Honduras, Guatemala, Costa Rica, Belize, El Salvador, and Panama. (DOCX)
