# Supplementary material for: Healthy competition drives success in results-based aid: Lessons from the Salud Mesoamérica Initiative
Source: PLoS One. 2017 Oct 27;12(10):e0187107. doi: 10.1371/journal.pone.0187107 (PMC5659784; doi:10.1371/journal.pone.0187107)
Supplement: S1 Text — (DOCX) [file pone.0187107.s001.docx]

**Annex 1: Topic guide for first group participants**

**What is your current position?**

How long have you been in this position?

Can you describe your major tasks and responsibilities?

If current position is not related to SMI:
***You have been chosen as a key informant in regards to the Salud Mesoamerica Initiative.***

**What was your position vis-à-vis of SMI?**

How long have you held that position?

Can you describe your major tasks and responsibilities within that position?

**How and why has SMI been designed as a partnership?**

Who are the different actors for health in Mesoamerica in general, and vis-à-vis of SMI in particular?

Who participated in the original overall regional design of SMI? Who was consulted during this process?

What were the different steps that led to the current general design of SMI?

What were the reasons behind the adoption of the theory of change?

What were the reasons behind the adoption of the regional strategy?

Who had the last word for big decisions when designing SMI?

Has this changed over time?

Who participated in the design of the operation of SMI in Mexico? Who was consulted during this process?

Did your jurisdiction participate in the design of the operation of SMI in Chiapas? Who else participated in this process?

What were the different steps that led to the current design of the operation?

Which of these steps were useful and which were not?

Do you think the order of the different steps should have been different and if yes, how?

What were the reasons behind the adoption of the operational strategy?

Who had the last word for big decisions when designing the operation?

What are the goals and visions of SMI?

Can you describe what SMI's main components are?

What are your goals as a donor for SMI?

Why did X join this partnership?

What is the role of X in this partnership?

Has your role changed over time?

What else in this partnership has changed over time?

How do you compare your experience within this partnership to other partnerships?

**To what extent were the strategies of SMI relevant and well designed for the best interest of the country?**

What are the common strategies, objectives, and goals between SMI and Mexico’s National Health Plan?

Are the timelines aligned?

Did you assess the needs and the main bottlenecks towards health in Mexico, and in Chiapas particularly prior to selecting SMI strategies?

How was that done?

Are the selected strategies integrated and aimed at addressing the bottlenecks?

Were the realistic capacities of the country/State to implement the program and in particular in relation to human resources considered during the process of designing SMI?

Was there a rigorous evaluation of the specificities of the different regions of the country, and in particular Chiapas, in order to adapt the strategies for action to their own characteristics?

How were the interventions selected?

What are the key community interventions and why were they selected?

How were the interventions selected?

At the onset of SMI, were any strategies or interventions considered and later eliminated?

If so, why?

**To what extent were the strategies of SMI:**

Aligned with national health plans for the different countries in content and timeline?

Based on integrated strategies aimed at addressing the bottlenecks?

**Use of information**

What are the different uses of the information generated from SMI?

How do you use this information to make decisions?

Can you give an example?

Has the information from external evaluation been useful for you?

If yes, why? If no, why not?

What was the role of targets and indicators?

Do you know SMI’s payment indicators and do you agree with them?

Is there awareness and buy-in regarding the payment indicators among all stakeholders (from funders to policymakers, to providers)?

How realistic are the targets to attain?

How difficult is it to attain these targets?

**Regarding SMI Policy Dialogue Model:**

Has SMI created new discussions around policies or changed the conversation around policies?

Has the process of creating, approving, and implementing policies changed due to SMI and how?

How has SMI contributed to the visualization and prioritization of the poor in the policy-dialogue agenda at the national and regional level?

**Why was a regional model adopted?**

What are the advantages and what are the disadvantages of a regional model?

What are the positive components of a regional model?

Does the model facilitate the coordination of stakeholders at different levels/sectors? If so, how?

**Are there delays and bottlenecks in the availability of funds and financial flows, and at what levels?**

Have there been delays and bottlenecks in the availability of funds and financial flows since the beginning of SMI, and at what levels?

What are the causes and what has been done to address them?

What is the influence of administrative and financial procedures on the implementation of SMI?

To what extent has the management of your ministry proved to be reactive to the difficulties encountered?

**How satisfied do you feel with the current level of implementation and where things are with SMI?**

How satisfied do you feel with the current level of implementation and where things are with SMI?

To what extent have the activities contained in SMI’s plan been implemented as planned (quality, quantity and terms)?

How does X monitor the implementation of activities?

Is the communication between X and partners effective?

Have you changed your practices based on lessons coming from other (countries/jurisdictions/health facilities)?

Have you shared lessons learned with other (countries/jurisdictions/health facilities)?

To what extent is the community engaged in the implementation of activities?

Is there any role for community-based organizations in the implementation of SMI activities?

**What are the organizational and contextual factors (such as the administrative and financial procedures, as well as the coordination mechanisms in place, and the political and social situation in the region) that have influenced (positively or negatively) the implementation of activities?**

To what extent has the management of SMI proved to be reactive to the difficulties encountered?

To what extent were the commitment and the support provided by IDB and partners, both during the preparation phase, as well as the implementation, appropriate and sensitive to contextual changes?

Have donor setbacks impacted their participation in the Initiative?

**What is the role of IDB in implementation?**

What is the role of IDB in implementation?

To what extent is IDB’s role effective?

To what extent has IDB’s role affected the implementation of the activities and the efficiency of the program?

To what extent has IDB’s role allowed acceleration of implementation of the activities and improving the efficiency of the program?

**To what extent have the resources and activities been coordinated, tracked and reported to IDB and partners?**

How are monitoring and evaluation activities conducted, discussed between partners, and used to take corrective action?

What are the lessons learned?

What went well and what did not go as well?

What measures could be taken to improve the effectiveness of SMI?

What could be done differently to improve efficiency?

**To what extent were the expected outputs (quality of care, access, equity) of SMI achieved?**

To what extent were the expected outputs (supply, use of maternal and child services, immunization services, preventive nutrition services, and health information systems) of SMI achieved?

What are the contextual factors that might explain the degree of achievement for certain outputs?

To what extent can any results be attributed to SMI?

What components of SMI influenced outputs achieved or not achieved according to stakeholders?

What components of SMI had the highest impact on the achievement or not of results according to stakeholders?

How can successful interventions be sustained through incorporation in policies, budget and healthcare models?

**Technical assistance**

What has been the role of MSH in implementation?

What has been the added value of MSH in this role?

What has been the role of the coordination unit?

What has been the added value of the coordination unit in this role?

**Mexico has been an exceptional case in failing, then succeeding, to meet indicators. To what extent was the improvement plan for Mexico justified, well conducted and relevant?**

What were the main factors leading to the improvement plan?

What was the take of your agency, as a donor, on that situation?

What was the take of your agency, as an implementer, on that situation?

How has the process been initiated and led? What has been the role of the partners?

What did you, as X, have to change for the improvement plan?

What are the lessons learned?

What went well and what did not go as well?

Did the improvement plan take into account the lessons learned from the first operational plan?

**What do you think are the incentives that are driving the countries under this support?**

**To what extent have the resources and activities been coordinated, tracked and reported to IDB and partners?**

How are monitoring and evaluation activities conducted, discussed between partners, and used to take corrective action?

What measures could be taken to improve the effectiveness of SMI?

To what extent have the financial resources been used as planned?

To what extent have actual expenditures been aligned with the initial budget?

**To what extent have certain major activities been effective in reaching their own goals (supply, training, community implication, etc.), and what are the factors that could explain these results?**

**What is the contribution of SMI in the health system?**

What changed in the ways you conduct business at X?

Specifically in Mexico and Chiapas?

Was there a change in the decision-making mechanism before SMI and the current situation?

Can you describe the collaboration and alignment inside X before SMI and the current situation?

Do you think these changes could have happened without SMI?

Has there been any shift in positions, change in roles, or hiring of new actors?

What has changed in governance and management due to SMI?

What are the changes that happened to human resources due to SMI?

What are the changes that happened to logistics and medicines due to SMI?

What are the changes that happened to service provision due to SMI?

What are the changes that happened to information system and data usage due to SMI?

What changes have been introduced at the community level for delivery of services? How?

Are health care providers able to adapt to this new environment?

What is their perception of these changes and how do these perceptions affect their performance?

Has the introduction of new equipment improved their productivity?

Did the trainings they received improve their record-keeping skills, leading to an improved health information system?

Are health providers concerned with user satisfaction?

Has SMI contributed to the prioritization of patients’ and users’ needs in the health system? Can you provide details?

Cultural competency is identified by SMI as a major need for improved care of vulnerable and indigenous populations. Have healthcare provider behaviors or attitudes toward indigenous women changed?

**Has SMI supported you to become more responsive to the population needs?**

Has SMI supported Ministries of Health to become more responsive to their population needs?

How about responsiveness toward X?

Can you speak specifically to the Secretaria de Salud in Mexico and Chiapas?

Is there a difference in the population perception of health services provided by X before and after the implementation of SMI?

**How can successful interventions be replicated/scaled up?**

How do you define success for SMI in general and for Mexico specifically?

How are the achievements of SMI at different levels (national, State, and operational) sustainable from a financial and program-level point of view?

What could be done differently to ensure the sustainability of the gains and changes achieved through SMI?

How can successful interventions be sustained through incorporation in policies, budget and healthcare models?

What exit plans are in place to sustain the results achieved through SMI?

What resources might be required in the future?

**What have been the unintended consequences (positive and negative) of SMI?**

What were the spillover effects of SMI in the performance of the health system in non-SMI areas of Chiapas?

**To what extent has this type of support added value compared to other means of health financing in the region, and in Mexico/Chiapas in particular?**

What was the role of SMI to align funds and actions to achieve common development objectives? (Alignment of funds from loans and other donations; Alignment of interventions from other organizations)

Did SMI attract other funds?

Are SMI funds complementary to other funds?

Has SMI created opportunities for additional funding to Chiapas?

What are the limitations of risk measures undertaken and how are they applied?

Have these risk management measures had any positive or negative results?

Has SMI created savings/efficiencies making new resources available?

What was the purpose of complementarity of funds for activities by governments?

Do you think the RBA mechanism has led to any misreporting of information?

Is there an accounting system carried out at the operational level?

How are expenses monitored? Is there potential for theft?

Are individual facilities rewarded if they meet their goals, and if so, how?

What happens if they don't meet their goals?

**How satisfied are users with health services currently?**

Has this changed since before SMI?

What are the drivers of user satisfaction in Chiapas?

What was the perception and experience of beneficiaries with the voucher system?

**To what extent are women benefitting from the available resources?**

Are there cultural factors that inhibit their use of available health care services?

Do they trust modern medicine?

Do they have firm beliefs about who should provide them with care?

How do they perceive health?

Are morbidity and mortality part of these beliefs?
