# Supplementary material for: Healthy competition drives success in results-based aid: Lessons from the Salud Mesoamérica Initiative
Source: PLoS One. 2017 Oct 27;12(10):e0187107. doi: 10.1371/journal.pone.0187107 (PMC5659784; doi:10.1371/journal.pone.0187107)
Supplement: S3 Text — (DOCX) [file pone.0187107.s003.docx]

**Topic guide: Focus Group Discussion**

1. **What is your current position?**
   1. How long have you been in this position?
   2. Can you describe your major tasks and responsibilities?

If current position is not related to SMI: ***You have been chosen as a key informant in regards to the Salud Mesoamerica Initiative.***

1. **What was your position vis-à-vis of SMI?**
   1. How long have you held that position?
   2. Can you describe your major tasks and responsibilities within that position?
2. **How and why has SMI been designed as a partnership?**
   1. Who participated in the original overall regional design of SMI? Who was consulted during this process?
      1. Who had the last word for big decisions when designing the operation?
         1. Has this changed over time?
   2. Why did {your organization} join this partnership?
   3. How do you compare your experience within this partnership to other partnerships?
   4. What aspects of SMI are distinct from other initiatives?
3. **To what extent were the strategies of SMI relevant and well designed for the best interest of the country?**
   1. What are the common priorities between SMI and your national health priorities?
4. **Use of information**
   1. What are the different uses of the information generated from SMI?
   2. How do you use this information to make decisions?
      1. Can you give an example?
   3. Has the information from external evaluation been useful for you?
      1. If yes, why? If no, why not?
   4. What was the role of targets and indicators?
   5. How realistic are the targets to attain?
5. **Regarding SMI Policy Dialogue Model:**
   1. Has the process of creating, approving, and implementing policies changed due to SMI and how?
6. **How satisfied do you feel with the current level of implementation and where things are with SMI?**
   1. Has SMI helped you introduce interventions that would not have been implemented otherwise?
   2. Have you changed your practices based on lessons coming from other (countries/jurisdictions/health facilities) and from the previous stage of SMI?
   3. Have you shared lessons learned with other (countries/jurisdictions/health facilities)?
   4. To what extent has SMI proved to be adaptive to the difficulties encountered?
   5. To what extent were the commitment and the support provided by IDB and partners, both during the preparation phase, as well as the implementation, appropriate and sensitive to contextual changes?
7. **What has been the contribution of SMI in your country?**
   1. What has been the contribution of SMI in your country?
8. **What have been the unintended consequences (positive and negative) of SMI?**
   1. Were there effects of SMI in the performance of the health system in non-SMI areas?
9. **How can successful interventions be replicated/scaled up?**
   1. How do you define success for SMI in general?
   2. How can successful SMI interventions be replicated/scaled up?
   3. What could be done to ensure the sustainability of the gains and changes achieved through SMI?
   4. What resources might be required in the future?
10. **To what extent has this type of support added value compared to other means of health financing in the region, and in your country in particular?**
    1. To what extent has this type of support added value compared to other means of health financing in the region, and in your country in particular?
11. **Other messages**
    1. Are there any other messages that you would like to share?
